# A High Precision, Wireless Temperature Measurement System for Pervasive Computing Applications

**DOI:** 10.3390/s18103445

**Published:** 2018-10-13

**Authors:** Christos Goumopoulos

**Affiliations:** Information & Communication Systems Engineering Department, University of the Aegean, 83200 Samos, Greece; goumop@aegean.gr; Tel.: +30-694-580-8993

**Keywords:** wireless sensor, temperature measurement, precision, calibration, ZigBee, pervasive computing applications, uncertainty analysis

## Abstract

This paper describes the design and calibration of a highly accurate temperature measurement system for pervasive computing applications. A negative temperature coefficient (NTC) thermistor with high resistance tolerance is interfaced through a conditioning circuit to a 12-bit digital converter of a wireless microcontroller. The system is calibrated to minimize the effect of component uncertainties and achieves an accuracy of ±0.03 °C on average (±0.05 °C in worst cases) in a 5 °C to 45 °C range. The calibration process is based on a continuous temperature sweep, while calibration data are simultaneously logged to reduce the delays and cost of conventional calibration approaches. An uncertainty analysis is performed to support the validity of the reported performance results. The described approach for interfacing the thermistor to the hardware platform can be straightforwardly adjusted for different thermistors, temperature ranges/accuracy levels/resolutions, and voltage ranges. The low power communication combined with the energy consumption optimization adopted enable an operation to be autonomic for several months to years depending on the application’s measurement frequency requirements. The system cost is approximately $45 USD in components, while its design and compact size allow its integration with extended monitoring systems in various pervasive computing environments. The system has been thoroughly tested and validated in a field trial concerning a precision agriculture application and is currently used in a health monitoring application.

## 1. Introduction

Pervasive computing represents a new paradigm where the physical world is merged with the digital world by embedding information and communication technology (ICT) capabilities into everyday objects and environments. Pervasive computing applications depend on a range of wireless sensor nodes and networks of sensors that collect and transmit contextual information, such as environmental and physiological data, to base stations which is crucial in order to provide valuable services in real-time [1]. In this context, the knowledge of accurate temperature values is significant in various domains, such as smart buildings [2], medical research [3], smart agriculture [4], and industrial processes [5]. Accurate and continuous temperature measurement is considered critical in the food industry to certify safe products of the highest quality [6]. This is because, with the support of an accurate temperature monitoring system, many of the thermobacterium germs that develop in food (e.g., meat and poultry) can be eradicated [7], or diseases can be detected early [8]. Likewise, monitoring body temperature variations for long periods of time can provide valuable information on human health conditions. For example, body temperature variability is associated with certain kinds of insomnia [9], cognitive operations [10], and circadian rhythm detection [11].

Currently, there is an increasing number of applications in the fields mentioned above where even slight temperature changes at the level of a few tens of milliKelvin need to be captured either to dynamically update domain knowledge models supporting precise decision-making functionality or to avoid unacceptable errors [12]. In precision agriculture, for example, temperature measurements with uncertainties at the level of 0.05 °C are required to analyze collected data with machine learning algorithms that search for accurate domain knowledge in order to optimize resource usage [4]. Biomedical research based on the remote monitoring of patients to gather clinically useful information requires a similar level of accuracy for the continuous monitoring of slight variations in skin temperature to enable an instant quantitative evaluation of tissue thermal conductivity. This is associated with accurate determination of skin hydration as well as with the characterization of vasoconstriction and vasodilation operations [13]. Environmental research in terms of observatory networks for the study of physical and biological processes by combining forecasting models and remotely sensed data requires a level of accuracy of up to 0.01 °C in fields like marine environmental research [14] and oceanographic research [15]. Also, specific industrial applications in precision manufacturing require similar levels of accuracy [16]. Optical systems, for example, can suffer from thermal deformations, even with slight changes in temperature, that can result in intolerable errors in their pointing accuracy. Time measurement can be also affected by tiny temperature changes with unacceptable drift for reference clocks.

Continuous, accurate temperature measurement is challenging when performed in everyday environments. A wireless temperature sensor for pervasive computing applications has a number of requirements that must be fulfilled:

- autonomy;
- network scalability (some applications may require large number of nodes);
- diagnostics (a logic that identifies that a sensor may not be working well);
- small size;
- low power;
- high resolution;
- high accuracy, sometimes precision is enough especially if the relative temperature is required and not the absolute value;
- possibility for being wearable;
- withstanding harsh environments (e.g., in agriculture).

The development of wireless sensors has been the target of both the research and industrial communities in recent years. In the next paragraphs, we discuss related work on systems that accurately measure temperature signals with characteristics similar to the proposed work.

A high precision temperature measurement system based on a thermocouple sensor is described in [17]. The sensor is connected to an 8-bit microcontroller to correct temperature online using a ninth order polynomial with standard coefficients and a reference junction compensation measured with a pre-calibrated temperature to digital converter. The accuracy achieved after calibration is ±0.08 °C, meeting industrial applications requirements. However, the communication with the back-end system is not performed wirelessly but serially (RS-232C). The design of a wireless temperature sensor required to achieve low drift and thus, high stability over long time periods (5 mK/year), again in industrial environments, is reported in [18]. A negative temperature coefficient (NTC) thermistor is interfaced through a bridge circuit to a microcontroller, Bluetooth communication is used; however, a medium accuracy level of 0.1 °C over a 15–30 °C range is reported.

In the agricultural domain, temperature measurement is an indispensable part of portable monitoring systems, and measurements are provided either via general purpose weather stations or through specifically developed systems using off-the-shelf components [19]. An example of a low-cost wireless temperature measurement system developed to monitor all stages of winery production, from the vineyard to the bottle, is reported in [20]. An off-the-shelf digital thermometer is interfaced to a ZigBee node. After calibration, the accuracy of the system is reported to be 0.4–0.8 °C depending on the media measured (ambient or liquid).

In the medical domain, a portable device with two thermistor channels for measuring body temperature is reported in [21]. The device is intended for clinical practice use, achieving a resolution of 0.01 °C, an accuracy of ±0.2 °C, and an operation autonomy of up to 10 days using a measurement frequency of 0.02 Hz. Bluetooth communication is used for data downloading. Accuracy is achieved by using standard calibration curves and high precision resistors. A passive Radio-Frequency Identification (RFID) temperature sensor optimized for ultra-low power consumption using 0.35 μm Complementary Metal–Oxide–Semiconductor (CMOS) technology is proposed in [22]. The sensor communicates with a reader over a distance of 2 m to transmit the measurements with a resolution of 0.035 °C and an accuracy of ±0.1 °C over a 35–45 °C range which makes it appropriate for human body temperature monitoring.

Another wireless temperature sensor category is the chipless sensor which is based on passive tags that do not contain any semiconductor technology or power source but rely on radio-frequency backscattering techniques to transmit the temperature data over relatively short distances. These antenna/resonator temperature sensors are classified as semi-invasive sensors and are placed at the area of interest, but the transmitted measurements are acquired remotely. The sensor tag may consist of a ceramic, dielectric resonator placed on a metal sheet, as in [23]. Since the dielectric resonator permittivity depends on the temperature, the resonant frequency emitted by the tag encodes the temperature value. In a different approach, the sensor node consists of a microstrip patch antenna serving as the temperature sensing unit, while an ultra-wide band (UWB) transmitting/receiving antenna with a Reactive Impedance Surface ground plane transmits the data [24]. Such sensors are used especially for control and monitoring processes in harsh conditions and high temperatures where silicon-based sensors cannot operate [25]. Their accuracy may range from ±0.1 °C to ±1.5 °C [23,26].

The non-linearity characteristic of certain types of temperature sensors presents a challenge for achieving accurate measurements. Besides the typical use of look up tables and on-line linearization, the use of more complex methods such as artificial neural networks has been applied to alleviate this problem [27]; however, such solutions may be difficult to implement considering the limited resources (battery power, processing speed, and memory capacity) of typical wireless nodes and the long lifetime operation expected by the foreseen applications. Instead, we propose a systematic calibration process of the measured signal.

From the above discussion, a conclusion that is drawn is that either the proposed systems are accurate but not portable, or the solution may be wireless but the accuracy is medium. In this paper, the design and calibration of a highly accurate (±0.03 °C on average) and low cost temperature measurement system is described which can be exploited in various pervasive computing application domains with precise measuring requirements in a 5–45 °C range. A low cost, thermistor-based sensing system is interfaced to a microcontroller which employs the ZigBee protocol for data transmission. The low power communication combined with the energy consumption optimization adopted in the measuring system enable operation autonomy for several months to years using measurement rates ranging from 0.0033 Hz (agriculture domain) to 1 Hz (medical domain).

A considerable focus of this work was the calibration of the measuring system. A conventional thermometer calibration process is a time consuming and expensive task since it requires both the reference thermometer and the tested device to be at precisely the same temperature in order to achieve thermal equilibrium before data recording. This has to be repeated for several temperatures for a given measurement range, resulting in a total required time between several hours (1–2 h per temperature step) in oil-based bath systems to more than a day in oven-based calibration systems. The advantage is that the uncertainties of the calibration process are minimized and an accuracy level of 1 mK can be delivered using purpose-built calibration systems [28].

Since the targeted applications and design constraints of the proposed measurement system cannot afford the time and cost of conventional calibration methods, we follow a different calibration process in a tradeoff between cost and accuracy. This process is based on a continuous temperature sweep while calibration data are simultaneously logged, requiring about three hours to complete without depending on specialized equipment. To support the validity of the reported performance results, an uncertainty analysis is implemented concerning both the design stage and calibration stage of the measurement system. In this context, we attempt also to experimentally justify that the uncertainties of the temperature sweep calibration in terms of the thermal delays between the reference device and the characterized sensors are affordable for the targeted accuracy.

## 2. Materials and Methods

### 2.1. Background

Temperature measurement is based on various sensor types [29]. These are broadly categorized as contact and non-contact sensors. The former require physical contact with the entity to infer its temperature when the system is in thermal equilibrium, i.e., there is no heat flow between the sensor and the entity. Thermistors, thermocouples, resistance temperature devices (RTD), and silicon-based sensors (e.g., bipolar junction transistor sensors) are commonly used as contact sensors. The majority of non-contact sensors measure the infrared thermal radiant power emitted by an entity and the surrounding surface. Infrared thermometers lead this category with two main subdivisions, spot and area-measuring thermometers. Table 1 shows typical characteristics of the most common temperature sensors, although other electric devices (e.g., thermopiles and piezoelectric temperature sensors) and non-electric devices (e.g., bimetallic, chemical molecular change, and fluid expansion) exist [30].

For the kind of application we are interested in supporting, a thermistor-based thermometer is most appropriate due to its low cost, high sensitivity and thermal conduction, low thermal mass, and small size which allows for unobtrusive solutions. Thermistors have a typical accuracy level of ±0.1 °C; however, their non-linearity, expressed by Equations (1) and (2) (where parameters *B* and *R*_0_ are known from the sensor data sheets and *T*_0_ is a reference temperature, typically 25 °C/298.15 K), presents a challenge when seeking highly accurate solutions. Parameters *T*, *T*_0_, and *B* are expressed in Kelvin units. The approach developed to overcome this problem is explained next.

(1) $$T=\frac{B}{\ln\left(\frac{R_{th}}{R_{\infty }}\right)}$$

(2) $$R_{\infty }=R_{0}\cdot e^{\frac{-B}{T_{0}}}$$

### 2.2. Temperature Measurement System Design

Our measurement system uses an NTC thermistor (model MF51E103E3950 by Cantherm, Montreal, QC, Canada) with a nominal resistance of 10 kΩ at 25 °C (±0.5% resistance tolerance). A measurement takes 10 ms, and the results are the average of 50 ADC readings (5 kHz ADC sampling frequency). Table 2 summarizes the main technical characteristics of the thermistor. Excess current flowing through the thermistor will cause self-heating and will result in a difference in the temperature reading. The temperature variance is related to the dissipation constant (e.g., when the heat of the component is one tenth/hundredth of the dissipation constant, the temperature difference will be 0.1/0.01 °C).

**Figure 1:**
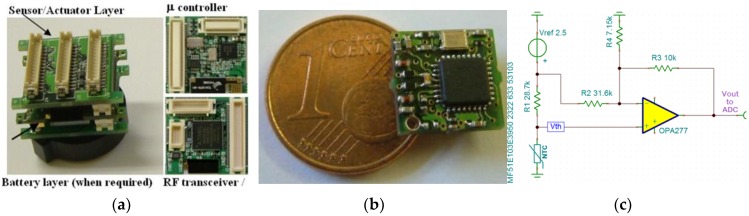
(**a**) Tyndall25 mote platform; (**b**) Tyndall10 mote; (**c**) thermistor interfacing circuit to the analog-to-digital converter (ADC).

The NTC thermistor is interfaced through a conditioning circuit to the analog-to-digital converter (ADC) module of Tyndall motes available in 25 mm and 10 mm form factors (Figure 1) [32]. Our hardware platform offers a 2.5 V reference voltage (*V_ref_*) which is obtained through a voltage divider (not shown in the circuit schematic) from the supply voltage that determines the maximum input voltage to the 12-bit ADC module. This makes the ADC count dependent only on the resistance values and eliminates any dependency on the tolerance of the ADC voltage reference. In this context, typically, three steps are involved to measure temperature values: (i) the ADC component measures the output voltage (*V_out_*) given by the thermistor interfacing circuit; (ii) the thermistor resistance (*R_th_*) is calculated as a function of the ADC value (*Count*); and (iii) the temperature is calculated by means of Equation (1). Table 3 gives the corresponding ADC values for indicative temperature values in the application range and related parameters in the conditioning circuit.

A high tolerance (0.1% tolerance) pullup resistor R1 is used in order to adjust the current that passes through the thermistor. This allows the self-heating of the thermistor to be controlled which affects the accuracy of the measurement. The temperature rise caused by self-heating represents a measurement error and this requires the *dissipation constant* of the thermistor to be examined. Given that we want an accuracy of at least 0.05 °C, the allowable dissipation would be 0.05 mW, but instead, by adding a safe margin to compensate for other errors and uncertainties in the system, we use 0.025 mW as the maximum allowable self-heating dissipation over the measurement temperature range. Given this bound and that we want to measure temperatures in the range of 5–45 °C using a 2.5 V voltage, the R1 resistor is calculated as follows. The thermistor resistance (from the data sheet) at 45 °C is 4356 Ω. A thermistor dissipation of 0.025 mW will result in a voltage drop over the sensor of 330 mV at 4356 Ω since *V* = Sqrt(*dissipation* × *Resistor*), and a current flowing of 75.75 μA. Consequently, the voltage drop across R1 will be 2.17 V and the minimum required resistance will be 28.7 KΩ (the nearest 0.1% value).

The conditioning circuit includes also a resistor network (R2, R3, R4) combined with an operational amplifier of high precision to scale and shift the thermistor voltage signal (*V_th_*). This allows for proper mapping of the *R_th_* range of interest (4.356 KΩ to 25.57 KΩ) to the microcontroller ADC voltage range (0 V to 2.5 V). It is necessary to scale the *V_th_* signal to get the required resolution. From the first and last rows in Table 3, we can see that the higher and lower *V_th_* values are 1178 mV and 329 mV, respectively. The *Count* for an output voltage (*V_out_*) in the measurement system is *V_out_*/*V_ref_* × 4096. So, the top and bottom counts (without scaling) are 1930 and 539, respectively. This means that for the range of temperatures we want to measure, there are 35 ADC counts [(1930 − 539)/(45 − 5)] per degree, which is less than the resolution we require (50 ADC counts for a 0.02 resolution)

The operational amplifier (op-amp) in the circuit provides the gain and the offset so that the output voltage matches the capabilities of the ADC module. Given that the op-amp is operating in the linear range (*V*_+_ = *V*_−_), the transfer function of the circuit is as described by Equations (3) and (4). By combining them and given that *V_out_*/*V_ref_* = *Count*/4096, a relationship between *R_th_* and the digital ADC output is obtained, as described by Equation (5):

(3) $$V_{out}=V_{th}\cdot \left(1+\frac{R3}{R4}+\frac{R3}{R2}\right)-V_{ref}\frac{R3}{R2}$$

(4) $$V_{th}=\frac{V_{ref}\cdot R_{th}}{R_{th}+R1}\;$$

(5) $$R_{th}=\frac{R1\cdot \frac{Count}{4096}+\frac{R1\cdot R3}{R2}}{1+\frac{R3}{R4}-\frac{Count}{4096}}$$

To select the appropriate values for R2, R3, and R4, the gain and the offset of the op-amp need to be calculated. First, the mapping of the temperature range of interest were designed to be between 0.1 V and 2.4 V at the ADC input, giving a little margin to compensate for system uncertainties. The higher and lower *V_th_* values were 1178 mV and 329 mV, respectively, which gave a Δ*V_th_* of 848 mV. The top and bottom ADC values (after scaling) were 3943 and 169, respectively (Table 3). This corresponds to 94 ADC counts per degree, satisfying our resolution requirements. Given that we designed the circuit so that the Δ*V_th_* swing of the thermistor output voltage is mapped into a swing of 2.3 V, this gave a gain of 2300/848 or 2.71. The offset can be found by subtracting the scaled higher or lower *V_th_* value (i.e., 1178 × 2.71 = 3192 mV) from the corresponding required voltage (i.e., 2400 mV). So, we calculated an offset of 792 mV or 0.79 V. By choosing R3 = 10 KΩ, we solved the equation system for the other two resistors, getting R2 = 31.52 KΩ and R4 = 7.18 KΩ (the nearest 0.1% standard values will be 31.6 KΩ and 7.15 KΩ, respectively).

Now, we can confirm that our measurement system offers the proper resolution for the designed temperature range. For NTC thermistors, the worst cases occur at higher temperatures. Using Table 3, we the sensitivity of the sensor is shown to be −30 mV/°C between 44 °C and 45 °C. Since the required temperature resolution was 0.02, the ADC voltage resolution needed to be at least 0.6 mV. The 12-bit ADC module of the Tyndall mote conveniently offers a resolution of 2.5/4096 = 0.6 mV.

### 2.3. Calibration

Equations (1) and (5) provide a complete model for temperature measurements from ADC outputs using the circuit in Figure 1c. However, the temperature accuracy depends on the tolerance of the circuit components and the thermistor’s B value given by the manufacturer. Therefore, the measuring system was calibrated to minimize the effect of element uncertainties and get highly accurate results. Equation (5) was rewritten as shown in Equation (6), where *ω* = *Count*/4096, *Κ* = *R*1, *L* = *R*1·*R*3/*R*2, and *M* = 1 + *R3*/*R4*. Moreover, Equation (6) was combined with Equation (1) to get the formula shown in Equation (7), where *K*′ = *K*/*R_∞_* and *L*′ = *L*/*R_∞_*:

(6) $$R_{th}=\frac{K\cdot \omega +L}{M-\omega }$$

(7) $$e^{\frac{B}{T}}=\frac{K^{\prime }\cdot \omega +L^{\prime }}{M-\omega }$$

Equation (7) is the calibration equation which relates the digital ADC value to the temperature. The main goal of the calibration process is to obtain a set of *T* and *ω* values that allows the calibration parameters *Κ*′, *L*′, and *M* to be computed. The advantage of this approach is that the entire circuit (sensor, resistors, op-amp, ADC) can be calibrated as a unit.

For the calibration process, we used a Tinsley 5885 high precision reference thermometer (0.001 °C resolution, ±0.01 °C accuracy, Tinsley, Essex, UK). We placed the thermometer and the thermistor in an insulated pot filled with water. The water was heated to reach a temperature of about 45 °C and then it cooled naturally while measurements are taken. Afterwards, the water was cooled to reach a temperature of about 5 °C and then it warmed naturally while measurements were taken. This process took more than 3 h as the temperature dropped by 0.01 °C every 3 s, and during this interval, the reference thermometer (*T_i_*) and the ADC (*ω_i_*) values were logged on a file, where each *ω_i_* entry was calculated by averaging 50 successive ADC readings. The file with the collected data was loaded to Matlab, and Equation (7) was used to fit the calibration data and finally, compute the unknown parameters using the least squares technique. The calibration values obtained were *K* = 1,627,697.417, *L* = 515,094.119, and *M* = 2.398. After plugging the calibration parameters into Equation (7), we were able to compute temperature values with high accuracy given an ADC value.

**Figure 2:**
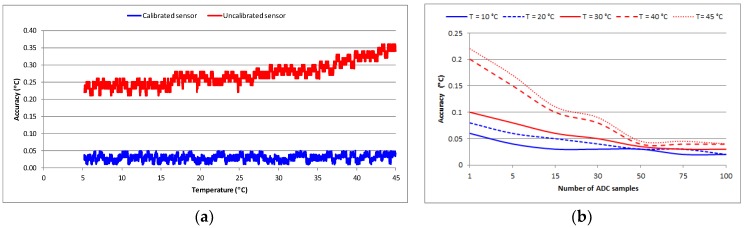
(**a**) Temperature sensor accuracy validation; (**b**) effect of ADC samples on the measurement accuracy.

Figure 2a shows the accuracy of the measurement system in absolute values. Without calibration we obtained an average error of ±0.26 °C for the temperature range of interest. In this case, Equations (1) and (5) as well as the parameters from the sensor data sheet were used to compute the temperature. The design of our circuit and the tight tolerance of the components used were shown to allow for a satisfactory accuracy level. When calibration was used, the average error was ±0.03 °C. In this case, Equation (7) and the computed calibration parameters were used to estimate the temperature. Calibration was shown to improve the temperature accuracy by, on average, approximately 9-fold. For comparison, the average error in cases where a linear approximation was used was ±1.2 °C.

As part of the calibration process, we also performed an analysis regarding the estimation of the number of samples required in order to reduce random noise during temperature measurements. For a specific temperature, the number of ADC samples was taken repeatedly and for each cycle, the average was computed as in the calibration process. The temperature value, as given by the calibration model (Equation (7)), was also computed in each cycle. The average temperature value was then compared to the real temperature which was given by the reference thermometer and the accuracy of the measurement was computed and recorded. Figure 2b illustrates the results of this analysis graphically. Using this information, the number of samples according to the requirements of an application can be chosen. Usually, there will be a tradeoff between the measurement accuracy and the energy efficiency. It is evident that in order to get an accuracy level of ±0.05 °C, 50 samples must be taken.

### 2.4. Uncertainty Analysis

Uncertainty in measurement is defined as the estimated probable deviation of a measurement result from its real value. The uncertainty of a measurement system is calculated by combining the uncertainties of its individual components using statistical methods. The two methods typically used are the combined standard uncertainty and the expanded uncertainty. The standard uncertainty is based on the uncertainty propagation law known as the root-sum-of-squares. The expanded uncertainty results from standard uncertainty via multiplication by a factor *k* which may be 2 or 3.

The standard uncertainty evaluation of the various components can be either Type A or Type B [33]. The former refers to a statistical analysis of observations (e.g., a series of measurements) whereas the latter refers to statistical measures (e.g., standard deviations from probability distributions) that are evaluated based on experience or other information about the measurement. For the uncertainty evaluation, a normal distribution (divisor 1) may be assumed when a probability distribution is formed via repeated measurements or when uncertainty reporting (e.g., given by device manufacturers, calibration certificates, etc.) is accompanied by the corresponding coverage factor. When there is no specific knowledge about the source of values, a rectangular probability distribution (divisor √3) is assumed where the values have an equal probability of lying within the stated interval [33].

#### 2.4.1. Design-Stage Uncertainty Evaluation

A zero-order uncertainty analysis combining Type A and B evaluations was conducted for the designed temperature measurement system to determine the combined standard measurement uncertainty using the root-sum-of-squares method. All the component uncertainties analyzed were assumed to be associated with independent variables of the measurement model (Equation (1) to Equation (7)) and parameters of the essential hardware. A first-order Taylor series approximation of the measurement model was used to compute the combined uncertainty related to the propagation of uncertainty law [33]. In general, if *y* = *f*(*x*_1_, *x*_2_, …, *x_n_*) is the measurement model of quantity *y* depending on *n* independent variables (*x*_1_, *x*_2_, …, *x_n_*), then the standard uncertainty of *y* is obtained by combining the standard uncertainties of the input estimates (*u_x_*_1_, *u_x_*_2_, …, *u_xn_*), as given by Equation (8), where the partial derivatives $\partial f/\partial x_{i}$ provide a sensitivity index related to the uncertainty of variable *x_i_*:

(8) $$u_{y}^{2}=\overset{N}{\underset{i=1}{\sum }}{\left(\frac{\partial f}{\partial x_{i}}\right)}^{2}u_{x_{i}}^{2}$$

The main variables required to compute *R_th_* (Equation (5)) are a voltage measurement by the ADC component (*Count*) and four resistor values (R1, R2, R3, R4) which are treated as variables in the domain of their nominal value variance. Therefore, the standard uncertainty related to these variables is either calculated or is taken from the manufacturers’ datasheets and propagated through Equation (1) to evaluate the combined standard uncertainty associated with the temperature measurement *T*.

For the voltage measurement by the ADC component, both Type A and Type B evaluations were used to determine the uncertainty (Table 4).

The combined standard uncertainty of the *R_th_* measurement is given by Equation (9), taking into account the uncertainties of the involved variables. A rectangular distribution was assigned to the tolerance of the resistors since the manufacturers’ specification limits were used as the uncertainty and these limits could not be traced from additional information [33].

(9) $$u_{R_{th}}^{2}={\left(\frac{\partial R_{th}}{\partial Count}u_{Count}\right)}^{2}+\overset{4}{\underset{i=1}{\sum }}{\left(\frac{\partial R_{th}}{\partial R_{i}}u_{R_{i}}\right)}^{2}$$

where $u_{R_{i}}$ = standard uncertainty of resistor *R_i_* (±0.1% resistor tolerance; normal distribution).

The open loop voltage gain (*A_OL_*) and the common-mode rejection ratio (*CMRR*) of the op-amp in the conditioning circuit may be the sources of gain errors in the measurement. Equation (10) gives the uncertainty of the op-amp due to *A_OL_* and *CMRR* [34]:

(10) $$u_{OA}=2\left(\frac{1}{\left|CMRR\right|}+\frac{1}{\left|A_{OL}\right|}\right)+\frac{R}{Z_{c}}$$

where *Z_c_* is the input impedance of the op-amp and *R* is the resistor value in the circuit model.

For the TI OPA277 component with *A_OL_* = 2 × 10^6^ (126 dB; minimum value), *CMRR* = 3.2 × 10^6^ (130 dB; minimum value), *Z_c_* = 250 GΩ (typical value), and *R* = 10 KΩ; the obtained value is $u_{OA}$ = 1.7 × 10^−6^. Given such a low value, we excluded this component from the following uncertainty analysis.

The combined standard uncertainty of the thermistor temperature measurement (Equation (11)) depends on the propagated uncertainty of the *R_th_* measurement and the tolerance of the sensor provided by the manufacturer.

(11) $$u_{T}^{2}={\left(\frac{\partial T}{\partial R_{th}}u_{R_{th}}\right)}^{2}+{\left(\frac{\partial T}{\partial R_{0}}u_{sensor,R0}\right)}^{2}+{\left(\frac{\partial T}{\partial B}u_{sensor,B}\right)}^{2}$$

where $u_{sensor}$ is the standard uncertainty of the sensor (±0.5% resistance tolerance; normal distribution, ±0.5% *B* value tolerance; normal distribution).

**Figure 3:**
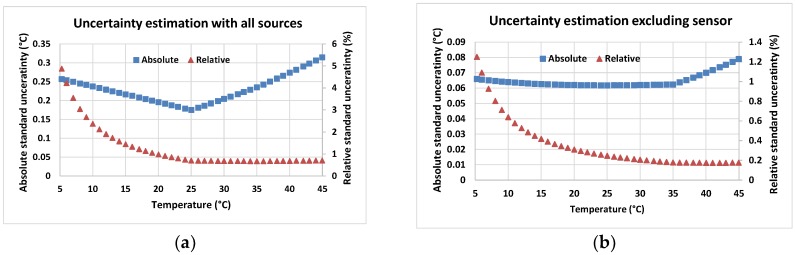
(**a**) Absolute and relative uncertainty estimation for temperature measurement based on system design and uncertainties of component nominal values; (**b**) uncertainty of the conditioning circuit components excluding the sensor tolerance.

By using Equation (11), the uncertainty of the temperature measurement system before calibration can be estimated based on uncertainties in component nominal values assessed through error propagation. Figure 3 shows the absolute and relative combined standard uncertainties related to the temperature measurement system. Figure 3a shows the total measurement uncertainty, taking into account all component uncertainties. Figure 3b shows the uncertainty of the conditioning circuit components excluding the sensor tolerance. The sensitivity analysis showed that, on average, the sensor tolerance contributes the most to the combined standard uncertainty (~70% or 0.16 °C), the resistor network contributes ~26.5% or 0.06 °C; and last is the ADC count (~3.5% or 0.008 °C). Since the sensor uncertainty dominates the relative contribution to $u_{T}$, the almost solid, absolute $u_{T}$ is sensible. The figures indicate that systematic errors exist which can be removed with appropriate calibration.

The actual average error of 0.26 °C in temperature measurement without calibration was shown to be slightly different to the estimated 0.23 °C by the uncertainty analysis. This difference can be attributed to other uncertainty sources such as the thermal noise from the resistors, measurement noise and the impact of equilibrium state which either were not considered or considered to be negligible in this analysis. If we had considered a rectangular distribution for the resistor and sensor tolerances, a choice that could have been justified by the fact that the provided uncertainties of nominal values have not been supported by coverage factors, then the estimated uncertainty would have been lowered to an average value of 0.13 °C, providing a larger margin for undercover uncertainties.

#### 2.4.2. Calibration Stage Uncertainty Evaluation

Thermistor calibration uncertainties are typically evaluated by following the instructions released by the International Committee for Weights and Measures in a guide that compiles the typical sources of uncertainty for thermometers based on NTC thermistors [35]. Based on the calibration process described in Section 2.3, the calibration-stage measurement uncertainty can be separated into two key parts: temperature measurement uncertainties and ADC count measurement uncertainties. The latter represents the standard uncertainty for voltage measurement by the ADC component which has been already discussed in the previous section in terms of the data given in Table 5. The temperature measurement uncertainties are associated with temperature readings by the reference thermometer and the continuous temperature sweep process involved. The uncertainty of the calibration model (Equation (7)) should be also taken into account in the form of the standard deviation of its error [33,36].

The reference thermometer used in the calibration process is a platinum resistance thermometer calibrated at fixed points with a standard uncertainty of 10 mK. Regarding the uncertainties of the temperature sweep calibration, an experiment was performed to examine the error margin caused by the shortage of perfect thermal stability and the temperature difference between the reference thermometer and the thermistor due to their different positions. Two thermistors calibrated with the model described previously were placed in a circulator water bath (Grant GD 120, stability ±0.05 °C, uniformity ±0.1 °C) for temperature measurement assessment against the reference thermometer. The thermistors and the reference thermometer were assessed at 5 temperature steps (5 °C, 15 °C, 25 °C, 35 °C, and 45 °C) at which measurements were taken only after the temperature in the water had balanced to within 25 mK for at least 20 min. The assumption was that if the continuous temperature sweeping time was adequate relative to the thermistor response time, then the measurement error in the relaxed steps would be comparable to the ones in the continuous temperature measurement.

**Figure 4:**
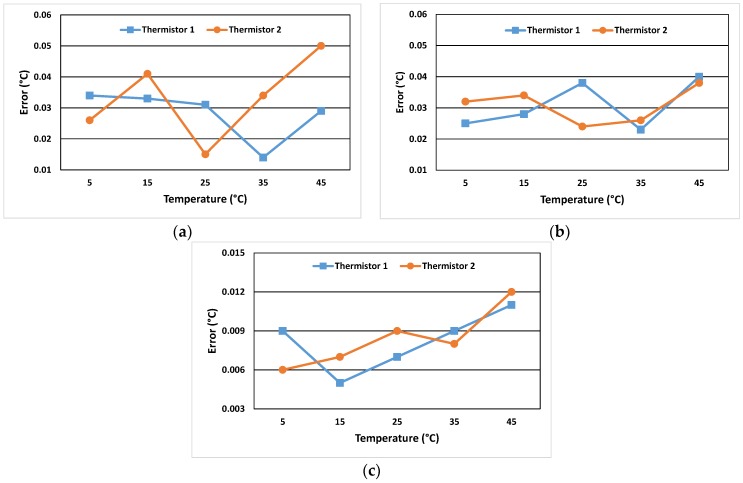
(**a**) Residual temperature offset of thermistors in the continuous temperature assessment; (**b**) residual temperature offset of thermistors in the step temperature assessment; (**c**) absolute values of errors between the two modes of temperature assessment.

Figure 4 shows the results. The spread of the errors in Figure 4a,b can be seen to have a random pattern which is a qualitative metric of the accuracy of the calibration model [36]. Apart from this, it is clear that the measurement errors are comparable and within the same accuracy limits. The step temperature assessment shows an improvement on the worst case error for both sensors (0.04 °C); however, the average remains the same (0.03 °C). Starting from high temperatures towards the lower ones, the continuous sweep shows a larger variation in measurement error between the sensors than the step temperature assessment. This indicates that it would be good practice to start the temperature sweeping higher than the current starting temperature. There is also a partial shift in the absolute error value of about 0.01 °C between Figure 4a,b which can be attributed to the stability of measurements in the step mode. However, this is not observed for all temperature values. As a result, the average error between the two modes of temperature assessment is 0.008 °C and its standard deviation is 2.3 × 10^−3^ °C which is considered to be the standard uncertainty of the temperature continuous sweep process. For the setup examined in this experiment, this error could be considered as being at the level of thermal noise of the sensors.

In order to verify the reproducibility of the results, a second evaluation was applied to the same sensors. The relative errors obtained were almost exactly the same as those obtained from the first test. This indicates that the systematic temperature offsets associated with the continuous calibration are constant.

The self-heating effects of the thermistor were also examined in the uncertainty analysis. According to [35] the self-heating uncertainty can be estimated by Equation (12), where *I* is the sensing current flowing through the thermistor, *R_th_*(*T_i_*) is the thermistor resistance at temperature *T_i_*, and *δ* is the dissipation constant of the thermistor:

(12) $$u_{th\_self\_heat}=\frac{I^{2}\cdot R_{th}\left(T_{i}\right)}{\delta }$$

Given the thermistor specifications in Table 2 (a dissipation constant of 2 mW/°C was used) and assuming a constant sensing current of 75.75 μA which results from the circuit design to compensate for the self-heating effect, we calculated the uncertainty of self-heating.

Finally, a source of uncertainty in the calibration process is the measurement noise caused by noise or variability in the measurement readings. This uncertainty was calculated as the experimental standard deviation of the mean.

In Table 5, the uncertainty budgets are provided for the calibration-stage analysis in the temperature range 5–45 °C. The worst case combined standard uncertainty (*k* = 1) for the calibration model presented was estimated to be 1.3 × 10^−2^ °C which is within the error margin assessed by the system evaluation.

### 2.5. Communication Protocol

The physical and the Medium Access Control (MAC) layers of the communication protocol employed by the wireless module are based on the IEEE 802.15.4 standard [37]. The upper layers of the protocol stack implement the ZigBee open specification [38] for the provision of short-range, low complexity, and low power communication which are appropriate for pervasive computing applications [39]. The IEEE 802.15.4 standard uses the 2.4 GHz frequency band and specifies a nominal transfer rate of 250 Kbps, supporting a transmission range from 10 to 100 m.

IEEE 802.15.4 combined with the ZigBee specifies networks with two kinds of devices designated as reduced functionality devices (RFD) and full functionality devices (FFD). Each device has a 64-bit IEEE address, even though short (16 bit) addresses can be used to reduce the packet size. In cases with short addresses, the payload of the packet is up to 114 bytes, whereas the maximum packet size is 127 bytes. A ZigBee network must have a coordinator which is responsible for network configuration, managing information exchanged in the network, and handling security keys. An FFD can be a network coordinator or a router. The latter acts as an intermediate, forwarding data between devices. An RFD is typically an inexpensive device (leaf node) that interacts with the physical world and has also the capability to interact with an FFD.

ZigBee supports three network topologies: star, mesh, and cluster tree. The star topology is particularly useful when the leaf nodes are closely clustered and can communicate with a single coordinator in a single-hop communication. Star configurations enable leaf nodes to implement power saving schemes and thus, to prolong the network’s lifetime. The mesh topology allows peer-to-peer interactions between adjacent nodes enabling multi-hop communications. The cluster tree topology is a combination of the preceding topologies where the root of the tree is allocated to the coordinator and all the non-leaf nodes are defined as routers which can forward the packets to/from the root.

The MAC layer of IEEE 802.15.4 operates in two different modes: non-beacon mode and beacon mode. In non-beacon mode, devices use the Carrier Sense Multiple Access with Collision Avoidance (CSMA/CA) physical layer protocol to access the radio channel; FFDs are always active ready to receive messages from RFDs, whereas the latter can stay in sleep mode for long periods of time. In beacon mode, all devices remain in sleep mode and they wake up only when a special packet (the beacon) is transmitted by the coordinator periodically using a slotted CSMA/CA protocol. The two operational modes dictate the power source requirements of the coordinator node: battery-source for beacon mode and mains-source for non-beacon mode.

The standard allows a maximum number of 10 hops (30 hops in ZigBee Pro release). The beacon operation mode allows for low energy consumption, even for large scale wireless sensor network applications, as discussed in [40]. Real-time requirements can be met in star topologies by using guaranteed time slots in the contention free period of the protocol’s superframe structure, whereas in mesh topologies, the delay of data transfer is affected by the number of hops. For applications with data transmitted at 5 min intervals, this may not be a problem; however, for industrial automation with real-time requirements, the average delays may be intolerable, requiring specialized MAC protocol solutions [41].

In the applications of our wireless temperature measurement system, the communication is limited to non-beacon mode and therefore, a star network topology is followed where the coordinator is mains-powered, while the sensor nodes are battery-powered. Besides simplicity, the star topology avoids several problems that have been acknowledged in the beacon operation mode, like FFD clock drifts in mesh and cluster-tree networks, network resynchronization due to the dynamic presence/absence of cluster nodes, and duty cycle reorganization when the coordinator fails.

### 2.6. Energy Efficiency

The wireless module contains an 8-bit microcontroller (Atmel ATMega 128L, Microchip Technology, San Jose, CA, USA) with 128 KB flash memory and a ZigBee transceiver (Chipcon CC2420, Texas Instruments, Dallas, TX, USA). Table 6 summarizes the typical current consumption of the main operations of the measuring system. The measurement frequency is configurable depending on the application’s needs and energy consumption restrictions. Considering a real-time monitoring case where a node includes one thermistor and a measurement is followed by a transfer—hence, both cycles are identical—the timestamped data packet transmitted wirelessly to the base station has a size of 21 bytes (6 bytes payload, 9 bytes MAC headers, and 6 bytes PHY headers) which corresponds to a 2.8 bps rate for a measurement cycle of 1 min. Other sensors can be integrated into the wireless module to measure additional parameters (e.g., humidity, light, air quality, etc.), increasing the data frame only a few more bytes and keeping the data rate low compared to the transfer rate of IEEE 802.15.4, i.e., 250 Kbps. This low data rate combined with a random sleep-wake pattern allows for the successful delivery of packets, even when a large number of motes is used in demanding applications.

When communication fails, the system keeps all the measurements in the local memory until the communication channel is restored. In case of memory overflow, new sensor measurements are suspended. In cases where the communication channel is temporarily unreliable resulting in unsuccessful packet transmissions, the interval between retransmission attempts at the application layer increases using a function that takes into account the number of failures and the measurement period in order to minimize energy waste.

In order to preserve energy, the wireless nodes enter the sleeping state whenever possible during their duty cycle. The latter is the ratio of the time to make and send one measurement to the measurement period. Assuming a measurement cycle of 5 min and averaging 15 samples per measurement (i.e., duty cycle of 0.07%), Table 7 summarizes the energy consumption for the various system operations. The receive operation is due to an acknowledgement frame which is required to consider the transmission successful.

The estimated average energy consumption is modelled by Equation (13):

*E_avg_* = *E_awake_* × *DutyCycle* + *E_sleep_* × (1 − *DutyCycle*) (13)

where *E_awake_* and *E_sleep_* denote the energy consumption during the active and sleep states, respectively. Given a scenario with an average energy consumption of 0.196 mW/0.157 mJ (Table 7), then an autonomous operation lifetime of 3 years is feasible if a 3.3 V Li-ion battery is used with 1200 mAh capacity (lifetime is estimated as *BattCapacity∙Voltage*/*E_avg_*). In cases with a higher measurement frequency, like 1 Hz, the duty cycle increases to 21.4%, and the average energy consumption is estimated to be 61.052 mW/1.087 mJ which leads to an autonomous operation of about 5 months.

### 2.7. Applications

**Figure 5:**
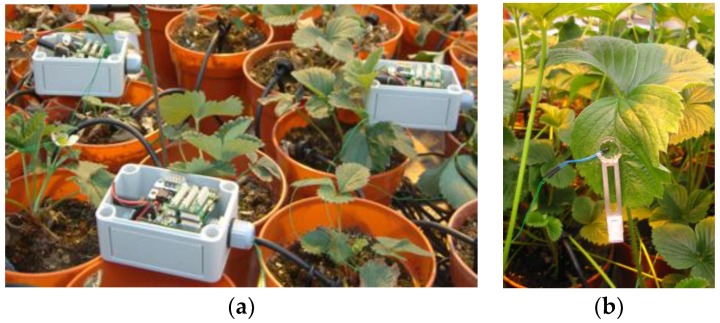

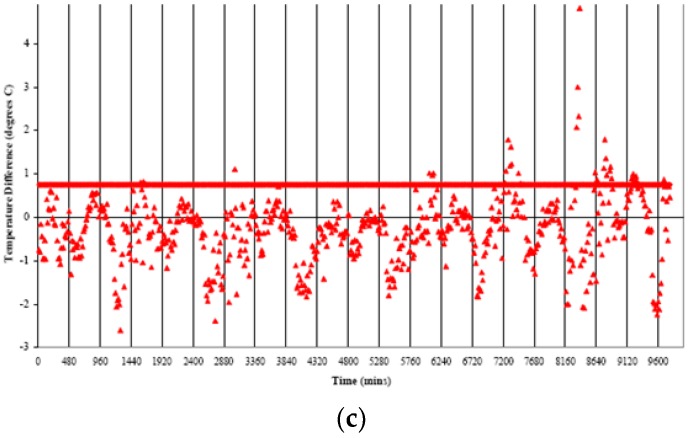
(**a**) Field trial of the system in a greenhouse; (**b**) a thermistor in contact with a strawberry leaf using a leaf clip; (**c**) temperature differences between a plant leaf and ambient air with a threshold of 0.84 °C to trigger water treatments.

The proposed measurement system was used in the context of an agriculture application that required plant leaf and environmental temperature measurements along with other parameters in order to decide precise irrigation treatments [4]. Furthermore, the system was required to support a machine learning process in order to induce new rules to improve the precision of plant state assessment in the decision-making process. In a greenhouse setting, a network of eight wireless modules including 36 thermistors was deployed and used for a period of 52 days providing accurate temperature measurements to the decision-making algorithm with a measurement period of 5 min (Figure 5a–c). The nodes were protected by water-resistant shielding to withstand the harsh field conditions. Accurate measurement of the temperature was a critical factor in the success of the derived agronomic model which resulted in a 20% irrigation water reduction compared to traditional practices while keeping the high quality of the crop.

The interface layer of the wireless microcontroller allows any combination of up to eight different sensors or actuators to be interfaced at any time and independently turned on and off. By interfacing several sensor devices (i.e., thermistors with their circuit) the number of modules required per sensor is reduced, which results in a lower overall cost. Each of the eight interface channel setups can easily be modified in the field in order to change the type of sensor/actuator attached. In this application, the deployment of the thermistors was a critical and time-consuming process. A series of leaf clips had been designed to attach thermistors to the plant leaves (Figure 5b). Thermistors were connected to the mote via two wires which had to be fixed to the bench top to minimize movement by the plant growth or by users. During the deployment, special care was given to choosing leaves that were of similar size, colour, shape, and location in order to minimize the local variations.

**Figure 6:**
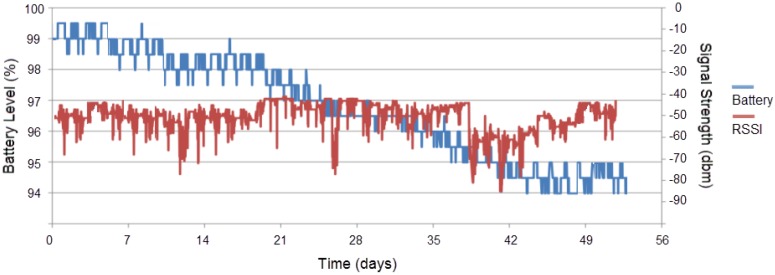
Battery level and signal strength readings for a period of 7.5 weeks.

A 3.6 V lithium thionyl chloride (LiSOCl_2_) battery with a capacity of 550 mAh was used (Tadiran TL-2450, Saft Groupe SA, Levallois-Perret, France) as the power source for the wireless nodes. This type of battery offers a high energy density and voltage stability over time while meeting the operating temperature range, capacity, and space requirements of pervasive computing applications. To monitor energy consumption, a reference unit was equipped with a CR2477 Li-coin cell battery (LiMnO_2_) with a capacity of 1000 mAh. The mote platform was also equipped with a voltage regulator (Torex XC6215, Torex Semiconductor, Tokyo, Japan) to provide stable voltage to system operations. Figure 6 shows the battery level and signal strength (Received Signal Strength Indication) plots during the trial period for the reference measuring node.

During the trial, the battery level dropped from 99% to 94% with a discharge of 0.1% or about 1.83 mAh per day. This confirms the assessment of the energy consumption albeit the small increase in the duty cycle to 0.11% due to the inclusion of four thermistors on the same unit. Most of the time, the signal strength was high (between 40 dbm and 55 dbm), except for some periods where there was high movement in the greenhouse due to labor activities. Our interpretation is that the presence of workers in the greenhouse affects the RSSI attenuation and variation due to the generation of multipath signal components. This observation is in accordance with studies that have shown that the presence of people moving in indoor environments significantly affects the signal strength, and the attenuation varies with the number of people, their location relative to the antennas, and their movement speed [42]. In the agricultural sector, energy harvesting through solar panels can significantly increase the autonomy of a wireless monitoring system, especially in open Southern European fields [43].

**Figure 7:**
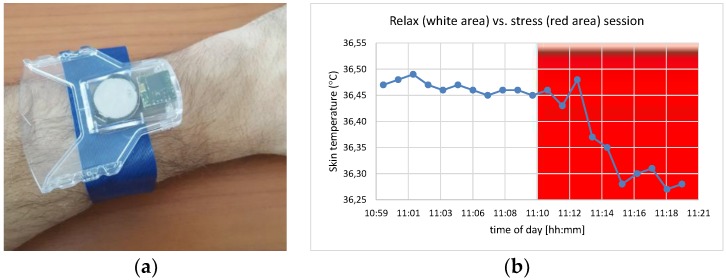
(**a**) Prototype package for health monitoring; (**b**) skin temperature variation between relaxation and stress periods.

We currently are using the developed system configured with the 10 mm mote in a health monitoring application that detects skin temperature in a non-intrusive manner (Figure 7a) and based on that detects user stress using a combination of physiological signals. The skin temperature is monitored with a frequency of 1 Hz and statistical features are calculated (mean, stdev, max, min) in different time windows. The Stoop test, which requires the participant to name the color of a word designated in a different color under time pressure, is used to induce mental stress to volunteers following an experimental protocol that includes relaxation and stress periods of 10 mins each, while the skin temperature signal variation is monitored (Figure 7b). Experiments with the prototyped wearable sensor have been performed in a laboratory setting with stable environmental conditions. However, in physical locations, the ambient temperature fluctuates during the day and this affects skin temperature measurements. To address this problem, differential models are used, taking into account both the skin and ambient temperature.

## 3. Discussion

The motivation for using a thermistor with high tolerance was to address the requirements of a multitude of applications for which a good accuracy (~±0.2 °C) could be achieved without turning to a specific calibration. The additional effort required for the calibration is justified for applications requiring higher accuracy levels as detailed in the introduction of the paper. Nevertheless, in other cases, a low tolerance and thus, lower cost thermistor can be used. The evaluation of the system proved that it is possible to reach a good accuracy without a specific calibration using components with a high tolerance. This observation was validated by the design stage uncertainty analysis discussed previously and is also consistent with other similar studies, such as in [21] where an accuracy of 0.2 °C was achieved by carefully designing a circuit with high precision components. In our case, a temperature error of 0.26 °C was observed.

Table 8 contains quantitative data for the assessment of the system’s performance in terms of the measurement error. The error is defined by Equation (14), where $T_{i}$ is the temperature measured by the reference thermometer, and $T_{i}^{*}$ is the temperature calculated by the system model (Equation (1) before calibration and Equation (7) after calibration). The minimum and maximum values of *e_i_* are denoted as *e_min_* and *e_max_*, respectively. A typical metric of the accuracy of a measurement system is the average error which is defined by Equation (15), where |*e_i_*| is the absolute value of *e_i_* and *n* is the amount of data. The smaller the average error is, the better the accuracy. The experimental standard deviation of the mean (Equation (16)) is an effective measure of the underlying probability distribution of the errors, where $\bar{e_{i}}$ is the average value of *e_i_*. It provides a measure of precision of the measurement system. The smaller the standard deviation is, the better the precision. This value is also used as a Type A uncertainty of the calibration equation measurement.

(14) $$e_{i}=T_{i}^{*}-T_{i}$$

(15) $${\left|e\right|}_{avg}=\frac{\sum \left|e_{i}\right|}{n}$$

(16) $$e_{std}={\left(\frac{\sum_{i=1}^{n}{\left(e_{i}-\bar{e_{i}}\right)}^{2}}{n-1}\right)}^{0.5}$$

The inspection of Table 8 shows that the accuracy of the measurement system has been improved by about 9-fold on average by using a calibration process which is different from conventional calibration approaches. The calibration process to reach the targeted accuracy is performed by correlating the ADC output of the interfaced measuring sensor with that of a reference sensor using a large set of temperature values. The continuous temperature sweep in about three hours makes the approach faster and cost effective with respect to conventional calibration procedures that are typically based on the use of specialized equipment. An additional advantage of the applied calibration is the continual evaluation of the sensor over the total specified range of temperatures. Estimating the calibration parameters for a collection of sensors in batch mode can reduce further the overall calibration time.

Although the wireless sensor network deployed for evaluating the temperature measurement system in the agriculture application used a limited number of sensor nodes and followed a star topology, more advanced schemes can be realized by leveraging ZigBee’s capabilities. Namely, by using the beacon operation mode of the ZigBee protocol, large scale deployments are feasible, covering larger areas and distances (hundreds of meters) between the sensor nodes and the base station through mesh networks. To cover even larger ranges (in thousands of meters), a solution proposed is the combination of ZigBee with 3G/4G cellular networks. This comes at the expense, however, of increasing energy consumption. The low power wide area network (LPWAN), an emerging wireless communication technology for Internet of Things (IoT) applications, stands as a promising solution for such large scales because of its low power, long range, and low cost communication characteristics [44]. LPWAN technology chipsets, like LoRa, are already in the market and are used in long range IoT applications. Given the modular nature of our wireless module, a LoRa transceiver could be integrated in the future.

Supporting high measurement accuracy and long sensor lifetime calls for a system design that assembles high precision and energy efficient components and the adoption of power consumption optimizations. As a result, a low average energy consumption in the region of 200 μW (as shown in Table 7) is achieved which, combined with the small-scale factor of the wireless platform, enables the system to be deployed in pervasive environments. The lifetime of the sensor module can be extended for demanding applications by using a battery with larger capacity or by connecting a number of batteries in parallel. Besides the energy harvesting already mentioned in the agricultural application, additional energy conservation can be brought into the system, for example, by dynamically adjusting the measurement period by taking context information (e.g., the presence of people in a greenhouse) into account.

A significant effort was devoted to the assembly of low-cost components, in order to realize a platform that could be integrated into extended monitoring systems. Based on an analysis of existing temperature sensors, the thermistor was selected because of its low cost and proper operational characteristics, and a conditioning circuit using off-the-shelf components was realized in order to create a low cost and portable thermometer that could be used in various domains. The cost of the sensing system is about $5 (includes the thermistor and the conditioning circuit components). The addition of the wireless board to support system intelligence and the ZigBee wireless connectivity adds an extra cost of $40. As a result of the design and choice of system parameters, the overall cost of the prototype is significantly less than the cost of laboratory thermometers with equal accuracy existing in the market (cost starts at $200).

Despite the low cost viewpoint, the measuring system was designed with the aim of identifying temperature-based physical and biological processes in pervasive computing environments with high accuracy. Table 9 provides a comparison between the characteristics of our measuring system and those of related systems reported in the literature as well as of sensors found in the market (last four entries) with the cost of components ranging from $15 (sensors without any wireless connectivity) to $60.

The limitations of this study are acknowledged. The evaluation of the system design and the calibration process were confined to the thermistors of a specific model. Although the steps of this methodology have been described in detail so that can be straightforwardly applied for different cases, a systematic validation is required using other sensors with different or similar characteristics with the used model. The design of the conditioning circuit attempts to control the self-heating of the thermistor which affects the accuracy of the measurement. Although the sensing currents allowed to pass through the thermistor are small, they can still generate self-heating errors. For example, thermal resistance caused by environmental temperature changes may vary due to air/liquid turbulence. This also affects self-heating error variation. Thus, the effect of temperature elevation on self-heating power needs to be addressed especially in the case of the outdoor health monitoring application. In the case of the agriculture application, the deployment was done in a greenhouse, and therefore, the measurements were performed in a more protected environment (e.g., physical shielding, light by low power fluorescent lamps, etc.). However, in general cases, measurement accuracy may be influenced by external factors such as air and thermal radiation, especially in outdoor environments; the parasitic effects of these factors need to be studied explicitly to determine a radiation correction model. Shielding of the thermometers (e.g., with a metal tube) may be also applied but care is required not to obstruct the air movement around the thermometers, otherwise the thermal contact between the air and the thermometer may be affected.

## 4. Conclusions

The design and calibration of a wireless autonomous thermometer that can provide continuous, real-time data to the decision-making mechanisms of various applications was described in this paper. By capitalizing on progress in ICT and wireless sensors, the proposed system addresses the challenge of developing a ubiquitous, robust, and autonomous low cost sensor while, at the same time, delivering measurements with high accuracy. To strike a balance between cost and accuracy, the calibration process follows a continuous temperature sweep approach to overcome the delays and cost of conventional calibration. This approach was shown to improve the accuracy of temperature measurement from ±0.26 °C to ±0.03 °C, on average. Furthermore, an uncertainty analysis was performed to support the validity of the reported performance results in which both the design-stage and calibration-stage of the measurement system were examined.

The developed temperature measurement system was evaluated in both lab and field settings for extensive periods of time, obtaining accurate values and transmitting them correctly using the ZigBee protocol to a base station. The low power communication combined with the energy consumption optimizations adopted in the measuring system enable an operation autonomy from several months to years depending on the required application measurement rates. The cost of the whole system is approximately $45 (related data are provided in Supplementary Materials) including the circuit components and wireless microcontroller, but excluding labor work for software development and testing which is affordable for practical use and allorews scaling of the number of sensing units in the same wireless module. In comparison, commercial thermistor-based systems of equal accuracy have a typical cost of $200 or more. The described procedure for interfacing the thermistor to the hardware platform can be straightforwardly adjusted for different thermistors, temperature ranges/accuracy levels/resolutions, and ADC voltage ranges.

Although high precision measurement systems may seem to have the scientific and industrial communities as their primary audience, we believe that as application intelligence and precise control become increasing necessities in pervasive computing environments, components which can deliver the accuracy of a laboratory thermometer with the cost of a common thermometer will be an important enhancement for many typical IoT applications. In this paper, we presented proof-of-concept applications that can take advantage of such progress.

## Figures and Tables

**Table 1:** Characteristics of temperature sensor types.

Characteristic	Thermistor	Thermocouple	RTD	Silicon	IR Thermometer
Range	−100 to 300 °C	−270 to 2300 °C	−200 to 850 °C	−45 to 125 °C	−40 to 3000 °C
Signal Output	Resistance	Voltage	Resistance	Resistance	Voltage
Linearity	Poor	Moderate	Best	Best	Moderate
Accuracy	Moderate (0.1 to 1.5 °C)	Low (0.5 to 5 °C)	High (0.03 to 1 °C)	Moderate (0.5 to 2 °C)	Low (±2 °C)
Sensitivity	Best	Low	Moderate	High	Moderate
Size (diameter)	0.4 to 2.5 mm	0.5 to 8 mm	3.17 to 6.35 mm	0.8 to 1 mm	Non-contact
Response Time	Moderate (0.1 to 10 s)	Moderate (0.1 to 10 s)	Slow (1 to 50 s)	Slow (5 to 50 s)	Fast (0.1 to 1 s)
Sensor/System Cost	Low/Moderate	Low/moderate	Moderate/high	Low/moderate	High/high
Advantages	High sensitivity; Small size; Copper/nickel wires; Low cost.	Self-powered; rugged; wide temperature range; interchangeable; no lead wire resistance problems.	Accuracy and stability; High repeatability; Interchangeable; Corrosion resistant.	Linearity and sensitivity; Low weight; very long operation life; energy efficiency.	No contact required; fast response; good stability; repeatability; no oxidation impact.
Disadvantages	Non-linear; Limited range; Self-heating; Current source required; Fragile; Specs and calibration vary by manufacturer; Lock-in due to lack of standards.	Non-linear; low output voltage; reference junction compensation required; lower accuracy; wire shielding is required; least sensitivity and stability.	High cost; slow response; low sensitivity; current source required; fragile.	Limited temperature range; highly non-linear at low/high temperatures; limited sizes; slow response time	High cost; complex electronics; view size restrictions; accuracy affected by object emissivity and background “noise” (smoke, dust, radiation).

**Table 2:** Thermistor specifications [31].

Dimensions		B Value (R25/50 °C)		Rated Power	Dissipation Constant	Thermal Time Constant
Lead	Wire	Nominal	Tolerance			
1.6 × 4 mm	0.2 mm	3950 K	±0.5%	3.5 mW	≥0.7 mW/°C	≤3.2 s

**Table 3:** Temperature values mapped to ADC values and related circuit parameters.

*T* (°C)	*R_th_* (ΚΩ)	*V_th_* (mV)	*V_out_* (mV)	Count
5	25.57	1178	2407	3943
5.58	25.17	1168	2380	3899
10	20	1027	1996	3270
15.3	15.61	881	1600	2621
20.38	12.32	751	1247	2043
25	10	646	963	1577
30.3	7.93	541	679	1112
35.98	6.25	447	422	691
40.64	5.17	381	244	400
44	4.52	340	133	217
45	4.356	329	103	169

*T* is the temperature; *R_th_* is the thermistor resistance; *V_th_* is the thermistor voltage; *V_out_* is the output voltage given by the thermistor interfacing circuit.

**Table 4:** Combined standard uncertainty for voltage measurement by the ADC component.

Source	Value (V)	Type	Probability Distribution	Standard Uncertainty (V)
Quantization error ^a^	3 × 10^−4^	B	Rectangular	1.7 × 10^−4^
Signal to distortion ratio ^b^	2 × 10^−4^	B	Rectangular	1.2 × 10^−4^
Repeatability ^c^	1.5 × 10^−4^	A	Normal	1.5 × 10^−4^
Combined standard uncertainty, *u_Count_*				2.5 × 10^−4^

^a^ ±0.5 LSB (least significant bit) Analog Devices AD7490 12-bit ADC resolution 0.6 mV BL^−1^; ^b^ 74 dB; ^c^ experimental standard deviation of the mean of 30 independent measurements of five constant voltages.

**Table 5:** Uncertainty budgets for the calibration stage.

Uncertainty Source	Type	5 °C [°C]	25 °C [°C]	45 °C [°C]
Reference thermometer calibration	B	1 × 10^−3^	2 × 10^−3^	5 × 10^−3^
Self-heating of thermistor	B	7.3 × 10^−8^	2.9 × 10^−8^	1.2 × 10^−8^
Calibration model interpolation error ^a^	A	8 × 10^−3^	8 × 10^−3^	8 × 10^−3^
Continuous temperature sweep error	A	2.3 × 10^−3^	2.3 × 10^−3^	2.3 × 10^−3^
Measurement noise of reference thermometer	A	7.3 × 10^−4^	7.9 × 10^−4^	8.1 × 10^−4^
ADC count measurement	A,B	8 × 10^−3^	8 × 10^−3^	8 × 10^−3^
Total combined standard uncertainty		1.2 × 10^−2^	1.2 × 10^−2^	1.3 × 10^−2^

^a^ See *e_std_* in Table 8.

**Table 6:** Current consumption of system operations (in mA).

Transmit	Receive	Sleep	Thermistor
17.4	19.7	0.001	0.08

**Table 7:** Energy consumption of system operations and average estimation, assuming a measurement frequency of 0.0033 Hz.

	Time (ms)	Current (mA)	Energy (mW/mJ)
Initialization	20	5.91	19.5/0.39
Transmit	35	40	132/4.62
Receive	0.5	40.45	133.5/0.07
Sleep	299,765	0.02	0.055/16.49
Thermistor	150	0.08	0.26/0.04
Avg.		0.06	0.196/0.157

**Table 8:** Quantitative metrics for the assessment of the measurement system’s performance.

	Before Calibration (°C)	After Calibration (°C)
*e_min_*	−0.21	−0.04
*e_max_*	0.36	0.05
|*e*|*_avg_*	0.26	0.03
*e_std_*	0.030	0.008

**Table 9:** Comparison of different temperature measurement sensors (the first entry refers to our system).

Sensor Type	Temperature Range (°C)	Accuracy (°C)/Resolution (°C)	Energy (mW) @ MF ^a^ (Hz)	Supply (V)	Connectivity	Ref
Thermistor	5 to 45	±0.03/0.02	0.196 @ 0.003	3–3.6	ZigBee	
Thermo-couple	0 to 200	±0.08/0.01	–	–	RS-232C	[17]
Thermistor	15 to 30	±0.1/0.002	1 @ 1	3.6	BLE	[18]
Silicon	0 to 60	±0.5/0.02	–	3	ZigBee	[20]
Thermistor	31 to 41	±0.2/0.01	1 @ 0.02	3.3	Bluetooth	[21]
Silicon	35 to 45	±0.1/0.035	110 × 10^−6^ @ 1	passive	Tag/Reader at 868 MHz	[22]
Antenna	40 to 100	±1.5 °C/–	–	passive	Tag/Reader at 5–6 GHz	[26]
Silicon	−40 to 125	±0.2/0.01	0.0032 @ 1	2.1–3.6	I^2^C	[45]
RTD	0 to 50	±0.5/0.1	3 yrs @ 0.001	3.6	WiFi	[46]
Silicon	10 to 50	±0.5/0.05	3 yrs @ 1	3	BLE	[47]
Thermo-couple	−40 to 85	±0.5/0.03	5 yrs @ 0.001	3.6	Probe/Reader at 20 KHz	[48]

^a^ Measurement frequency.

## Supplementary Materials

The following are available online at http://www.mdpi.com/1424-8220/18/10/3445/s1, Table S1: BOM for the wireless temperature measurement system (prices given for orders of 1, 100 and 1000 units). Table S2: Assembly cost estimation (price ranges are per board for orders of 5, 100 and 1000 boards).
