# Supplementary material for: A High Precision, Wireless Temperature Measurement System for Pervasive Computing Applications
Source: Sensors (Basel). 2018 Oct 13;18(10):3445. doi: 10.3390/s18103445 (PMC6210438; doi:10.3390/s18103445)
Supplement: Supplementary file 1 [file sensors-18-03445-s001.pdf]

Supplementary Material

A High Precision, Wireless Temperature Measurement System for Pervasive Computing Applications

Christos Goumopoulos

Hardware system cost estimation

The Bill of Material (BOM) with prices for the components integrated in the wireless temperature measurement system is given in Table S1 (price quotes have been acquired from [1]).

Table S1. BOM for the wireless temperature measurement system (prices given for orders of 1, 100 and 1000 units).

Component	Function	Unit Cost (1) USD	Unit Cost (100) USD	Unit Cost (1000) USD
Atmel ATMega 128L 8AU	Microcontroller unit	8.3	7.0	7.0
Chipcon CC2420RGZR	ZigBee transceiver	3.1	3.1	3.1
Torex XC6215	Voltage regulator	0.2	0.2	0.2
Tadiran TL-2450	Battery	3.8	3.0	2.4
AD7490BRUZ-REEL	12-bit ADC	5.8	5.8	5.8
MF51E103E3950	NTC thermistor	2.2	1.3	1.0
OPA277PAG4	Operational amplifier	1.5	1.5	1.5
Vishay CMF55xxKxxxBERE	4 Resistors (0.1% tolerance)	0.8	0.8	0.8
Sum		25.7	22.7	21.8

Low cost Printed Circuit Board (PCB) manufacturing is possible since the wireless board design has quite low design constraints of 0.15 mm track-size and gap-size, and 0.3 mm drill size for vias [2]. Furthermore all the required components are commercially available (Table S1), to keep costs low, and to have low lead times for manufacturing the boards. Assembly companies typically manufacture PCBs based on the design plans received from the client and their prices depend on parameters such as board quantity, size and thickness, number of layers, lead time and shipping country. Online market research indicates that PCBs have a price of approximately 0.5-1 USD/inch^2 for order quantities of at least 100 pieces [3]. Given the system design characteristics and the 25mm x 25mm form factor of the wireless mote, a PCB cost of 0.20 USD is possible (assuming 100 units). A price of 5 USD will be quoted for a minimum order quantity of 5 PCBs.

Assembly costs can be also estimated using parametric on-line quote calculators based on the number of PCBs, number of unique parts (BOM lines), number of fine pitch and leadless parts on each board, number of through holes, assembly sides etc. [3], [4]. Table S2 provides an assembly cost estimation of the wireless temperature sensor using the on-line quote calculator in [3].

Table S2. Assembly cost estimation (price ranges are per board for orders of 5, 100 and 1000 boards).

Assembly Cost (5) USD	Assembly Cost (100) USD	Assembly Cost (1000) USD
74–93	9.36–18.8	5.85–11.78

We have estimated that the hardware cost of the whole system will be approximately 45 USD when a minimum of 40 PCBs can be ordered.

References

1. Usa-Global-Chips.com, https://www.usa-global-chips.com/
2. Harte S, O'Flynn B, Martínez-Català RV, Popovici EM. Wireless sensor node design for heterogeneous networks. Proceedings of the 32nd International Conference on Microelectronics and Packaging. 2008.
3. PCBShopper.com, https://pcbshopper.com
4. bittele PCB Assembly, http://www.7pcb.com/PCB-Assembly-Quote.php
